# Characterization of lipid parameters in diabetes mellitus – a Nigerian report

**DOI:** 10.1186/1755-7682-2-19

**Published:** 2009-07-20

**Authors:** Anthonia O Ogbera, Olufemi A Fasanmade, Sonny Chinenye, Akinyele Akinlade

**Affiliations:** 1 Department of Medicine, Lagos State University Teaching Hospital, Ikeja, Lagos, Nigeria; 2 Department of Medicine, General Hospital Gbagada, Lagos, Nigeria; 3 Department of Medicine, Lagos University Teaching Hospital, Idi-araba, Lagos, Nigeria; 4 Department of Medicine, University of Port-Harcourt Teaching Hospital, Port-Harcourt, Nigeria

## Abstract

**Background:**

Diabetes mellitus (DM) is a disorder that is often associated with cardiovascular events and underlying lipid abnormalities. Cardiovascular complications are common causes of DM deaths in Nigeria yet dyslipidaemia is one aspect of DM that is underdiagnosed and undertreated in our patients. This report seeks to determine the prevalence and pattern of lipid abnormalities in Nigerians with types I and 2 DM.

**Methods:**

A total of 600 patients with DM aged between 22 – 79 years were evaluated for lipid abnormalities. The anthropometric indices, glycosylated haemoglobin, pattern of DM treatment and co-morbidities were noted. Total cholesterol (TCHOL), triglyceride (TG), high density lipoproteins (HDL-C), low density lipoproteins cholesterol (LDL-C) levels and the atherogenic indices levels were documented. Test statistic used included student's t test and χ^2^.

**Results:**

Well over half (89%) of the study subjects had lipid abnormalities and there was no statistically significant difference in the proportions of subjects with type 1 and 2 DM with lipid abnormalities. Elevated LDL-C, TCHOL, TG and reduced HDL-C were noted in 74%, 42%, 13%, and 53% respectively of the study subjects. The commonly noted combined lipid abnormalities were elevated TG and reduced HDL-C. Hypertension, significant histories of smoking and alcohol ingestion were found to be potential determinants of the occurrence of dyslipidaemia. Age, sex, type of DM and anthropometric indices were found to be determinants of the the pattern of dyslipidaemia. Only a small proportion – (8%)-of the subjects with dyslipidaemia were on treatment for it.

**Conclusion:**

Having defined the scope of dyslipidaemia in our patients and also highlighting its gross undertreatment, we hope that our data will help sensitize health care practitioners on screening for and treating dyslipidaemia. Elevated LDL-C and reduced HDL-C should be the primary targets of treatment in our patients with dyslipidaemia.

## Background

Diabetes mellitus is one of the most important non communicable diseases in Nigeria and is second only to hypertension in terms of public health significance. The high burden of DM in Nigeria is largely attributable to cardiovascular diseases which account for 15% of all DM deaths [1,2]. The development of cardiovascular disease in DM is often predicted by several factors which include central obesity, hypertriglyceridemia, elevated low high-density lipoprotein (HDL-C) levels, and hypertension [3]. Hypertriglyceremia and low high-density liopoproteinaemia are two components of the atherogenic profile seen in DM. Elevated low density lipoprotein (LDL-C) has also been found to be an independent risk factor for the development of cardiovascular disease and is often reported to be the commonest lipid abnormality found in patients with DM [4-6]. The presence of elevated cholesterol levels is known to play a key role in both the initiation and progression of atherosclerosis, as well as in the clinical consequences such as myocardial infarction, stroke, peripheral vascular disease, and heart failure [7]. Hypercholesterolemia has also been implicated in the process of atherogenesis and a curvilinear relationship has been documented between increasing cholesterol and increasing incidence of CVD [7]. The role of LDL-C in the development of CVD cannot be overemphasized as there is documented evidence that high levels of LDL-C not only cause atherosclerosis but pharmacological interventions that reduce LDL-C cholesterol are associated with stabilization and regression of atherosclerosis in proportion to the cholesterol lowering achieved[8]. Low levels of HDL-C have been consistently reported in cardiovascular diseases [9-11]. Although the triglycerides have been found to be univariate predictors of CVD in many studies, no clinical trial data has established that lowering triglycerides in individuals with or without diabetes independently leads to lower CVD event rates after changes in HDL cholesterol are adjusted for.

From the foregoing, it is evident that elevated cholesterol, low HDL, high TG and high LDL are all risk factors for CVD. The pattern of occurrence of these abnormalities in type 2 DM especially has been severally reported in both developed and developing economies [11-13].

The majority of previous studies on the lipid profile of patients with DM in Nigeria were carried out in patients with type 2 DM. In this report we undertake to determine the pattern of lipid abnormalities and clinical correlates in patients with types 1 and 2 DM.

## Methods

This is a cross sectional study that featured 600 subjects with DM drawn from the General hospital Gbagada and the Lagos State University Teaching hospital Ikeja. These are the two main government owned hospitals that serve the large majority of people in the South Western region of Nigeria. The study subjects were recruited over a period of eight months. All consenting adults with DM were recruited for the study after ethic clearance was given by the Ethics committees of both hospitals.

All study subjects were given interviewer administered questionnaires that included information on biodata, duration and treatment type of DM. The presence of hypertension and its treatment type, history of present/prior usage of the statins or other lipid lowering drugs.

All subjects were weighed with their foot wear off and the weight was rounded to the nearest 0.1 kg and the body mass indices (BMI) were calculated. Waist circumferences were determined by applying a tape measure to the midpoint between the inferior margin of the last rib and the crest of the ilium. Blood pressure measurement was done with a mercury sphygmomanometer.

### Laboratory tests

Fasting blood samples were taken for the determination of 4 parameters of the lipid profile viz total cholesterol (TCHOL), high density lipoprotein cholesterol (HDL-C), and triglyceride (TG). Total cholesterol assay was done using a modified method of Liebermann-Burchard [14], HDL-cholesterol by precipitation method [15] and TG was estimated using a kit employing enzymatic hydrolysis of TG with lipases [16]. LDL-C was calculated using the Friedwald's formula [17] LDL = (TCHOL - HDL-C) - TG/5 when the values of TG were less than 400 mg%.

### Operational definitions

1. Type 2 DM-Patients were classified as having type 2 diabetes mellitus using clinical criteria such as a present/prior history of usage of oral hypoglycaemic agents or usage of combination of insulin and the oral hypoglycaemic agents.

2. Type 1DM- This referred to patients who are presently on insulin and have been insulin requiring since diagnosis.

3. Dyslipidaemia: Abnormal lipid profile consists of the following abnormalities either singly or in combination. These include triglyceride (TG) levels ≥ 150 mg%, high density lipoprotein cholesterol (HDL-C) (for men ≤ 40 mg% and women ≤ 50 mg%), low density lipoprotein cholesterol (LDL-C) ≥ 100 mg% [9,18,19].

Also considered abnormal is an elevated total cholesterol level ≥ 200 mg% [18].

4. Poor glycaemic control refers to glycosylated hemoglobin (Hba1c) levels of ≥ 7% [17].

5. Atherogenic indices refer to TCHOL/HDL-C and LDL-C/HDL-C ratios

The TCHOL/HDL-C and LDL-C/HDL-C ratios were calculated and a ratio of >4.

5 was considered abnormal for men and ratios of below >4 abnormal for women [20].

6. Age cadre: For purposes of this study, patients were classified according to their ages into three age groups viz young, middle aged and elderly. Young age group referred to those who were less than 40 years of age, middle aged to those ≥ 40–65 and elderly which is >65 years of age.

Statistical analyses were performed with SPSS version 15. Independent sample Student's t test was used to compare quantitative data. χ^2 ^was used to test for differences in proportions. A p value of ≤ 0.05 was deemed significant.

## Results

### Clinical characteristics of the study subjects

The mean (SD) age and age range of the study subjects was 58(10) years and 22–79 years respectively. Subjects with type 1 DM made up 15 (2%) of the study subjects. Females were 399 (66%) and Males 201(34%). The BMI for the study subjects ranged 14.8–56.9.7 k/m^2^. Significant alcohol and smoking history were noted in 61 (10%) and 138 (23%) respectively of the study subjects. Well over half 354(59%) of the subjects had a history of hypertension. The mean duration of DM was 6.5(6.3) with a range of 0.1–40 years. The mean glycosylated haemoglobin was 6.8 (2,2) years and the range was 3.6–18%.

The comparison between the clinical features of types 1 and 2 DM are shown in Table 1.

**Table 1:** Comparison of some clinical parameters between subjects with type 1 DM and those with type 2 DM

**Clinical parameters**	**Type 1 DM**	**Type 2 DM**	**P value**
Duration of DM	8.6 (12.2)	6.5 (6.1)	0.2

BMI	27.4 (7)	28.8 (5.9)	0.3

WC	91.2(19.2)	95.1 (13.6)	0.2

Age	34.2(13.5)	58.7(9.9)	0.0001

A1c	6.6(3.9)	6.8(2.4)	0.8

∫Htn	4(27%)	350(60%)	0.000001

∫-hypertension

#### Pharmacologic therapies in the study subjects

The oral hypoglycaemic agents were used by 490 (81.7%), Insulin by 39 (6.5%), a combination of insulin and oral hypoglycaemic agents by 62(10.3%) and sole dietary management of DM by 9 (1.5%) of the subjects. Only 42 (8%) of the subjects with dyslipidaemia were on treatment with statins. Antihypertensive agents used included thiazides, calcium channel blockers alpha methyl dopa and the beta blockers.

### Lipid abnormalities

The percentage of lipid abnormalities was 89% (524 of the subjects had varying degrees of lipid abnormalities). The percentages of elevated LDL-C, elevated total cholesterol, reduced HDL-C, and elevated TG were 74%, 42% 53%, and 13% respectively. The least difference noted between the mean values of the lipid parameters in those with and those without dyslipidaemia was in the triglyceride values. These results are shown in table 2.

**Table 2:** Mean fasting lipid profiles in subjects with and those without abnormal lipid values

Variable	Dyslipid	Non-dyslipid	Mean difference	P value
TChol	198(45)	158(22.8)	39.9	0.0000001

HDL	42.7(17.8)	64.8(19.2)	-22.1	0.00000001

LDL	136.8(46)	74(18.9)	62.7	0.00000001

TG	99.9(49.3)	84.4(29)	15.9	0.00007

Anthropometric indices and other clinical parameters did not differ between the study subjects with abnormal and those without abnormal lipid parameters. These results are shown in table 3. Hypertension was present in 354 (59%) of the study subjects. The ratio of subjects with abnormal lipid profile and hypertension to that of those with normal lipid profile with hypertension is 314:40. This difference in the proportions of these study subjects with hypertension is significant (p = 0.00001). The proportion of subjects with dyslipidaemia who smoked was greater than that of those that did not have dyslipidaemia and this difference was statistically significant (50(82%) vs 11 (12%) p = 0.0000001). The proportion of subjects with dyslipidaemia who had a significant alcohol history was greater than that of those that did not dyslipidaemia and this difference was also statistically significant (115(83%) vs 23 (17%) p = 0.00001).

**Table 3:** Comparison of some quantitative parameters between DM subjects with abnormal and those with normal lipid profiles

**Clinical parameters**	**Dyslipid**	**Non-dyslipid**	**P value**
Duration of DM	6.6(6.2)	6.5(7)	0.9

BMI	28.8(5.9)	28.2(6.3)	0.3

WC	95.5(13.1)	93(13.4)	0.3

Age	58(10)	57.2(12.8)	0.4

A1c	6.8(2.4)	6.7(2.6)	0.8

Of the combined lipid abnormalities the combination of elevated TG and reduced HDL-C was the prevalent abnormality as this was detected in 37 (7%) of the subjects. The combination of elevated TG and LDL-C- was documented in 25(5%) and the combination of elevated TG, LDL-C and low HDL-C was found in 14(3%) of the study subjects. The combination of elevated TCHOL, TG, LDL-C and low HDL was the least documented lipid abnormality.

Gender distribution of lipid abnormalities showed that 357 (89%) females and 165 (83%) males had varying degrees of abnormal lipid profile. However there was no statistical significance in the gender distribution of lipid abnormalities. (p = 0.89). There were however notable gender differences in some of the components of the lipid parameters. These are shown in table 4.

**Table 4:** Blood lipid values for study subjects and gender comparisons

Variables	Males	Females	P value
TCHOL	182.7(40.2)	198.2(47)	0.0001

HDL-C	44.1(8.7)	46.2(19.8)	0.2

LDL-C	117.6 (41.2)	134.4(50.7)	0.0002

TG	96.2(49.2)	99.1(46.6)	0.4

TCHOL/HDL-C	4.9(2.8)	4.9(2.3)	0.9

LDL-C/HDL-C	3.3(2.5)	3.4 (2.2)	0.5

A total of 337 (62%) of the study subjects had abnormal TCHOL/HDL-C ratios. The proportion of women with abnormal TChol/HDL-C ratio was higher than that of men and this difference was statistically significant (236 (70%) vs 101(30%) p = 0.000001). Abnormal LDL-C/HDL-C ratios were documented in 156 (26%) of the study subjects. As the TCHOL/HDL-C ratios, abnormal LDL-C/HDL-C ratios were noted more in females than in males and this difference was statistically significant (112(72%) vs 43(28%) p = .000001).

The prevalence of abnormal lipid profile was higher in type 2 DM- 517(87%) than type 1 DM-11(73%) but this difference was not statistically significant. (p = 0.09). Three lipid parameters (TG, TCHOL/HDL-C and LDL-C/HDL-C) differed significantly between types 1 and 2 DM. The comparisons in lipid parameters between types 1 and 2 DM are shown in table 5

**Table 5:** Distribution of lipid profile and HbA1c amongst subjects with types 1 and 2 DM

Variables	Type 1DM	Type 2DM	P value
TChol	192.1(43.3)	193(45.4)	0.9

HDL	51(12.1)	45(19.5)	0.2

LDL	121.5 (47.5)	124.4(48.6)	0.5

TG	80.6.(26.7)	98.4(47.8)	0.02

TCHOL/HDL-C	3.9 (1.3)	4.9 (2.5)	0.01

LDL-C/HDL-C	2.5(1.2)	3.4 (2.3)	0.02

A1c	6.6(3.9)	6.8(2.4)	0.8

### Body Composition and lipid profile

**Figure 1:**
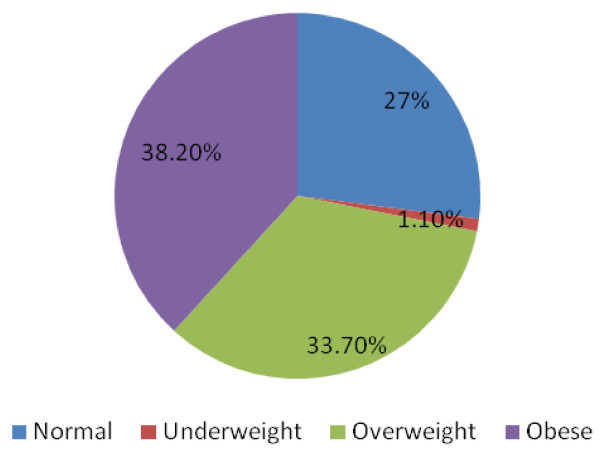
Distribution of study subjects by BMI.

Well over half of the study subjects were overweight/obese. 229(38.2%). The BMI distribution of the study subjects is shown in Figure 1.

Well over half of the study subjects with dyslipidaemia were overweight/obese. 176 (34%) of the subjects were overweight and 207 (40%) were obese. All study subjects who were underweight had lipid abnormalities which occurred singly or in combination. These results are displayed in Table 6.

**Table 6:** Pattern of lipid abnormalities associated with BMI groups

Clinical parameters	HDL	TG	LDL	TCHOL
Normal	76(24%)	12(16%)	111(25%)	66(26%)

Underweight	2(1%)	2(1%)	7(7%)	5(3%)

Overweight	102(32%)	25(33%)	153 (35%)	84(33%)

Obese	138(43%)	37(49%)	170(39%)	96(38%)

### Distribution of abnormal lipid profile amongst the different cadre of age groups

Middle aged, elderly and the young made up 247 (42%), 317 (52%) and 36 (6%) respectively of the study population. Triglyceride was the only lipid parameter that differed significantly in the three age groups. The comparisons are shown in table 7

**Table 7:** Age group differences in lipid parameters

Variable	Elderly	Middle aged	Young	P value
TChol	79(44%)	161 (42%)	11(31%)	0.3

HDL-C	98(54%)	205(53%)	15(41%)	0.3

LDL-C	129 (71%)	286(74%)	26 (72%)	0.7

TG	18 (10%)	56(15%)	1(2%)	0.05

## Discussion

Diabetes mellitus is often associated with cardiovascular morbidity and this may partly be explained by the abnormal lipid profile which is sometimes a feature of DM. With heart failure and cerebrovascular disease increasingly becoming prevalent in our population, it is imperative to determine possible risk factors accounting for this scenario. One such risk factor of note is an abnormal lipid profile.

We undertook to study the pattern of lipid abnormalities in a relatively large population of DM subjects with those with types 1 and 2 DM inclusive. Type 1 DM not surprisingly made up 2% of the study population and differed significantly from type 2 DM in age and the presence of hypertension. The percentage of lipid abnormalities occurring singly or in combination at 89% was high and this was comparable to that reported in a recent Nigerian study [21] which gave a figure of 89%. The magnitude of the detected abnormalities showed that LDL and TCHOL were the parameters that were most affected as they showed the greatest mean differences between the values in those that had abnormal and those with normal lipid profiles. Elevated cholesterol though not usually regarded as highly predictive of cardiovascular disease was noted in 42% of the participants The significance of screening for total cholesterol lies in the fact that is could serve as a valuable screening measure for dyslipidaemia. The aforestated scenario not withstanding, we report elevated LDL-C and reduced HDL as the prevalent lipid abnormalities in our study. Similar findings have been noted in a recent Nigerian report [21] and that carried out in an African-American population [22]. A Kenyan report [13] on the lipid abnormalities in DM had elevated cholesterol and LDL as the commonest lipid abnormalities noted in their study.

Although we report a higher prevalence of lipid abnormalities in type 2 DM than type 1DM this difference was not significant. A Western report[23] on lipid abnormalities in DM noted a significantly higher prevalence of lipid abnormalities in type 2 DM compared to type 1 DM. The presence of hypertension may however be contributory to the greater prevalence of dyslipidaemia in type 2 DM than in type 1 DM. The relationship between insulin resistance or compensatory hyperinsulinaemia may partly explain the aforestated scenario [24]. Insulin resistance often leads to increased intracellular hydrolysis of triglycerides and release of fatty acids into the circulation and the resultant inability of fat cells to store triglyceride is the initial step in the development of dyslipidaemia. Other plausible explanations for the contributory effect of hypertension to abnormal lipid profiles in DM include usage of antihypertensive agents [25]. The beta blockers and diuretic especially the thiazide diuretics have been found to negatively affect not only the lipid profile but glucose tolerance. Of these two blood pressure lowering agents, the thiazide diuretics were commonly used for the purpose of blood pressure control in our study subjects with DM and hypertension. The pattern of lipid abnormalities in types 1 and 2 differed with respect to triglyceride, TCHOL/HDL-C and LDL-C/HDL-C ratio which were all significantly higher in type 2 DM than type 1DM thus implying a higher cardiometabolic risk in subjects with type 2 DM. Although the habits of smoking cigarettes and ingesting alcohol were not widely practiced by our patients, significant alcohol and smoking histories were found to be contributory to the occurrence of dyslipidaemia.

We found that clinical parameters like age, sex, duration of DM, waist circumference, BMI type of DM and glycaemic control were not possible determinants of the presence of an abnormal lipid profile. However, sex was found to be a possible determinant of the pattern of lipid profile levels in diabetes. Other factors that were found to affect the pattern of lipid abnormalities included age and BMI. In this report, we noted that elevated TCHOL and LDL-C were found to be significantly higher in females than in males and HDL differed significantly between subjects who were underweight and all other cadre of BMI. Cook et al [22] in their report on gender differences in pattern of dyslipdaemia noted that elevated LDL-C and reduced HDL-C were more commonly documented in females than males. Bowden et al [26] found gender differences in the HDL-C, LDL-C and TG components of the lipid profile in non -diabetic individuals. From the foregoing it is evident that gender differences are consistently noted in LDL-C in individuals with and those without DM. Although the mean values of the atherogenic indices were comparable in both sexes, the proportion of women with abnormal atherogenic indices was significantly higher than men.

Elevated triglyceride was more significantly elevated in the middle and elderly age group than in the younger age group. The prevalent combination of lipid abnormalities was that of elevated TG and reduced HDL, two defining parameters of the metabolic syndrome [16]. These two lipid abnormalities are the most commonly noted abnormalities of the standard lipid profile in subjects with obesity and insulin-resistance-related cardiometabolic risk.

We have showed that lipid abnormalities are underdiagnosed in our patients with DM as despite a documented high prevalence of dyslipidaemia only 8% of affected individuals were on treatment.

## Conclusion

The prevalence of lipid abnormalities in our patients with DM is unacceptably high and only few people with these abnormalities are on treatment. Reduced HDL-C and elevated LDL-C are the prevalent lipid abnormalities in our patients with DM. Although hypertension, significant alcohol ingestion and smoking histories are possible determinants of dyslipidaemia in our report, gender, age, type of DM and anthropometric indices are observed to affect the pattern of occurrence lipid abnormalities.

## Competing interests

The authors declare that they have no competing interests.

## Authors' contributions

AOO designed the study, participated in data collation, statistical analysis, funding and writing the draft of the manuscript.

OAF participated in data collation, funding and writing the draft of the manuscript.

SC participated in funding, and statistical analysis.

AA assisted in data collation.

## References

[B1] Ogbera AO (2007). Burden of Diabetes mellitus in Nigeria. Trop Doct.

[B2] Ogbera AO, Chineneye S, Onyekwere A, Fasanmade O (2007). Prognostic Indices of DM mortality. Ethn and Disease.

[B3] Sumner AE (2008). The relationship of body fat to metabolic disease: influence of sex and ethnicity. Gend Med.

[B4] Nesto RW (2005). Beyond low-density lipoprotein: addressing the atherogenic lipid triad in type 2 diabetes mellitus and the metabolic syndrome. Am J Cardiovasc Drugs.

[B5] Udawat H, Goyal RK (2001). Lipid lowering effect of simvastatin in patients of type 2 DM. Indian Heart J.

[B6] Idogun ES, Unuigbe EP, Ogunro PS, Akinola OI, Famodu AA (2007). Assessment of serum lipids in Nigerians with type 2 diabetes mellitus complications. Pak J Med Sci.

[B7] Brunzell JD, Davidson M, Furberg CD, Goldberg RB, Howard BV, Stein J, Witztum JL (2008). Lipoprotein management in patients with cardiometabolic risk. Consensus statement from the American Diabetes Association and the American college of Cardiology Foundation. Diabetes Care.

[B8] O'Keefe JH, Cordain L, Harris WH, Moe RM, Vogel R (2004). Optimal low-density lipoprotein is 50 to 70 mg/dl: lower is better and physiologically normal. J Am Coll Cardiol.

[B9] Sani-Bello F, Bakari AG, Anumah FE (2007). Dyslipidaemia in persons with type 2 diabetes mellitus in Kaduna, Nigeria. Int J Diabetes and Metabolism.

[B10] Singh IM, Shishehbor DO, Ansell BJ (2007). High-density lipoprotein as a therapeutic target: a systematic review. JAMA.

[B11] Idogun ES, Unuigbe EP, Ogunro PS, Akinola OI, Famodu AA (2007). Assessment of serum lipids in Nigerians with type 2 diabetes mellitus complications. Pak J Med Sci.

[B12] Williams K, Tchernof A, Hunt KJ, Wagenknecht LE, Haffner MS, Sniderman AD (2008). Diabetes, abdominal adiposity and atherogenic dyslipoproteinaemia in women compared with men. Diabetes.

[B13] Otieno CF, Mwendwa FW, Vaghela V, Ogola EN, Amayo EO (2005). Lipid profile of ambulatory patients with type 2 diabetes mellitus at Kenyatta National Hospital, Nairobi. East Afr Med J.

[B14] Abell LL, Levy BB, Brodie BB, Kendall FE (1952). Simplified methods for the estimation of the total cholesterol in serum and demonstration of specificity. J Biol Chem.

[B15] Lopez-Virella ML (1977). Cholesterol determination in high-density lipoproteins separated by three different methods. Clin Chem.

[B16] Bucolo G, David H (1973). Quantitative determination of serum triglycerides by the use of enzymes. Clin Chem.

[B17] Friedwald WT, Levy RI, Fredrickson DS (1972). Estimation of the concentration of low density lipoprotein cholesterol in plasma, without use of the preparative ultra centrifuge. Clin Chem.

[B18] American Diabetes Association (2009). Standards of Medical Care in Diabetes-2009. Diabetes Care.

[B19] Alberti KGMM IDF Consensus on the metabolic syndrome: Definition and treatment. http://www.idf.org/webcast.

[B20] Cholesterol ratio information. http://www.medicinenet.com/artasp.

[B21] Okafor CI, Fasanmade OA, Oke DA (2008). Pattern of dyslipidaemia among patients with type 2 diabetes mellitus. Niger J Clin Pract.

[B22] Cook CB, Erdman DM, Ryan GJ, Greenland KJ, Giles WH, Gallina DL, El-Kebbi MI, Ziemer DC, Ernst KL, Dunbar VG, Phillips LS (2000). The pattern of dyslipidaemia among African-Americans with type 2 diabetes. Diabetes Care.

[B23] Perez A, Wagner AM, Carreras C, Gimenez G, Sanchez-Queseda JL, Rigla M (2000). Prevalence and phenotypic distribution of dysipidaemia in type 1 diabetes mellitus. Arch intern Med.

[B24] Salonen JT, Lakka TA, Lakka AM, Valkonen VP, Everson SA, Kaplan GA (1998). Hyperinsulinaemia is associated with the incidence of hypertension an dyslipidaemia in middle aged men. Diabetes.

[B25] Ferrari P, Rosner J, Weidmzn P (1991). Antihypertensive agents serum lipoproteins and glucose metabolism. Am J Cardiology.

[B26] Bowden R Lipid levels in a cohort of sedentary university students. The internet journal of cardiovascular research.

